# Cross-Species Interaction between Rapidly Evolving Telomere-Specific *Drosophila* Proteins

**DOI:** 10.1371/journal.pone.0142771

**Published:** 2015-11-13

**Authors:** Balázs Vedelek, András Blastyák, Imre M. Boros

**Affiliations:** 1 Department of Biochemistry and Molecular Biology, University of Szeged, Szeged, Hungary; 2 Institute of Biochemistry, Biological Research Centre of the Hungarian Academy of Sciences, Szeged, Hungary; St. Georges University of London, UNITED KINGDOM

## Abstract

Telomere integrity in *Drosophila melanogaster* is maintained by a putative multisubunit complex called terminin that is believed to act in analogy to the mammalian shelterin complex in protecting chromosome ends from being recognized as sites of DNA damage. The five proteins supposed to form the terminin complex are HP1-ORC associated protein, HP1-HOAP interacting protein, Verrocchio, Drosophila Telomere Loss/Modigliani and Heterochromatic Protein 1. Four of these proteins evolve rapidly within the *Drosophila* genus. The accelerated evolution of terminin components may indicate the involvement of these proteins in the process by which new species arise, as the resulting divergence of terminin proteins might prevent hybrid formation, thus driving speciation. However, terminin is not an experimentally proven entity, and no biochemical studies have been performed to investigate its assembly and action in detail. Motivated by these facts in order to initiate biochemical studies on terminin function, we attempted to reconstitute terminin by co-expressing its subunits in bacteria and investigated the possible role of the fast-evolving parts of terminin components in complex assembly. Our results suggest formation of stable subcomplexes of terminin, but not of the whole complex *in vitro*. We found that the accelerated evolution is restricted to definable regions of terminin components, and that the divergence of *D*. *melanogaster* Drosophila Telomere Loss and *D*. *yakuba* Verrocchio proteins does not preclude their stable interaction.

## Introduction

The ends of the linear genetic material represent two problems regarding their faithful maintenance throughout cell generations. First there is the problem of end replication by the replicative DNA polymerases, which can result in a gradual loss of genetic material during replication cycles [1, 2]. The second problem is that chromosome ends might be recognized as double-stranded DNA breaks (DSB) that can trigger DSB repair, resulting in structural rearrangements of chromosomes and keeping checkpoint processes sustained at the expense of suspending normal cell cycle [3]. These problems have been circumvented during eukaryotic evolution by the “invention” of the telomere and its associated proteins.

Chromosome ends are usually elongated by telomerase through reverse transcription that results in repetitive telomeric DNA [4]. Telomere ‘capping’ proteins bind these repetitive DNA sequences to form a protecting ‘cap’ complex. It has been proposed that the single-stranded part of the telomere (3’-overhang) folds back to its homologous sequence and hybridizes to its complement while displacing the identical strand. This DNA structure is called a t-loop [5]; it prevents the end from being recognized by components of DNA repair and checkpoint processes as a double-stranded DNA break [6, 7].

In *Drosophila* both the elongation of the chromosome ends and the inhibition of the chromosome fusions follow a seemingly different way compared to human and other canonical telomeres [8, 9, 10]. Drosophila chromosome ends are elongated by insertions of non-LTR retrotransposons such as HeT-A, TART and TAHRE, instead of reverse transcription by telomerase [11, 12, 13]. Therefore it is not surprising that in the lack of short telomeric repeats of defined sequences, the “canonical” capping proteins are also missing. Nevertheless, capping must take place and it has been suggested to be performed by a complex [14] consisting of the HP1-ORC associated protein (HOAP) [15, 16], the HP1-HOAP interacting protein (HipHop) [17], the Verrocchio (Ver) [18], the Drosophila Telomere Loss (DTL) also known as Modigliani (Moi) [19, 20] and the conserved Heterochromatic Protein 1 (HP1) [21, 22, 23]. Immunostaining verified that these proteins co-localize at the telomeres, and deletion of any of the genes encoding these proteins causes chromosome fusions [14, 16, 17, 18, 19, 21]. In addition, interactions between individual members of this alleged protein complex were also demonstrated by GST pull-down experiments [17, 18, 19]. Further studies revealed that HOAP and HipHop are even capable to mutually stabilize each other at the telomeres [17]. The above-described evidences indicate that these proteins most probably participate in the same pathway and contribute to telomere maintenance.

Similarly to t-loops in canonical telomeres, *Drosophila* telomeres are also postulated to be complex structures containing both double- and single-stranded DNA [11]. This assumption and the observations on DNA-binding properties of some terminin members suggest the possibility of their interaction with DNA as a multivalent entity.

Based on these observations, the existence of a multisubunit protein complex has been suggested that may work in analogy to the canonical capping complex. It has been designated as the *Drosophila* terminin complex [14]. However, the lack of purified material precluded biochemical characterization of the putative complex and particularly its DNA-binding affinity. Moreover, even the very existence of the terminin complex as a discrete entity needs further verification.

A particularly interesting feature of most of the terminin proteins is their accelerated evolution. Comparing *D*. *melanogaster* proteins with their orthologs from other *Drosophila* species revealed that HOAP, HipHop, Ver and DTL/Moi are more diverse in their amino acid sequence than *Drosophila* proteins on average [14, 15, 17–19]. This surely raises some concerns regarding complex assembly. Do the changes occur in an essentially random pattern or do they affect only discrete parts of the proteins? In case of this latter possibility, the fast-evolving parts may mark distinct functional domains of the proteins. Such domains can be, for example, interaction motifs which should change concomitantly within each interacting partner to ensure proper interplay. On the other hand, it can be expected that if the changes hinder the formation of molecular interactions between terminin components of closely related but distinct species, thereby these ultimately contribute to post-zygotic isolation.

In order to initiate studies addressing these questions we analyzed the pattern of rapidly changing residues in terminin subunits, and found that they define discrete parts of the proteins, which can be considered as domains in most cases. Next we tried to reconstitute the terminin complex by expressing its components in a heterologous system. We found that four out of the five terminin members can be expressed at high level in bacteria but form insoluble aggregates. Co-expression improved protein solubility; however, we detected the formation of only two discrete subcomplexes, despite that previous data are compatible with the existence of a stable heterotetrameric subcomplex of terminin [18, 19]. We used one of the subcomplexes, the stable Ver-DTL/Moi heterodimer to address the possibility of interspecies heterodimer formation, and found that formation of such a dimeric structure between *D*. *melanogaster* and *D*. *yakuba* proteins can in fact occur.

## Results

### Accelerated evolution affects discrete parts of terminin proteins

The speed of evolution is usually quantified by the proportion of non-synonymous (pN) and synonymous (pS) substitutions. Higher pN/pS ratio means faster evolution of a protein. Based on this criterion *Drosophila* telomere capping proteins with the exception of HP1 show accelerated evolution [14, 15, 17, 18]. However, pN/pS values are statistical, thus reflect the evolution rate for entire molecules, though that could be significantly different within molecules.

**Fig 1:**
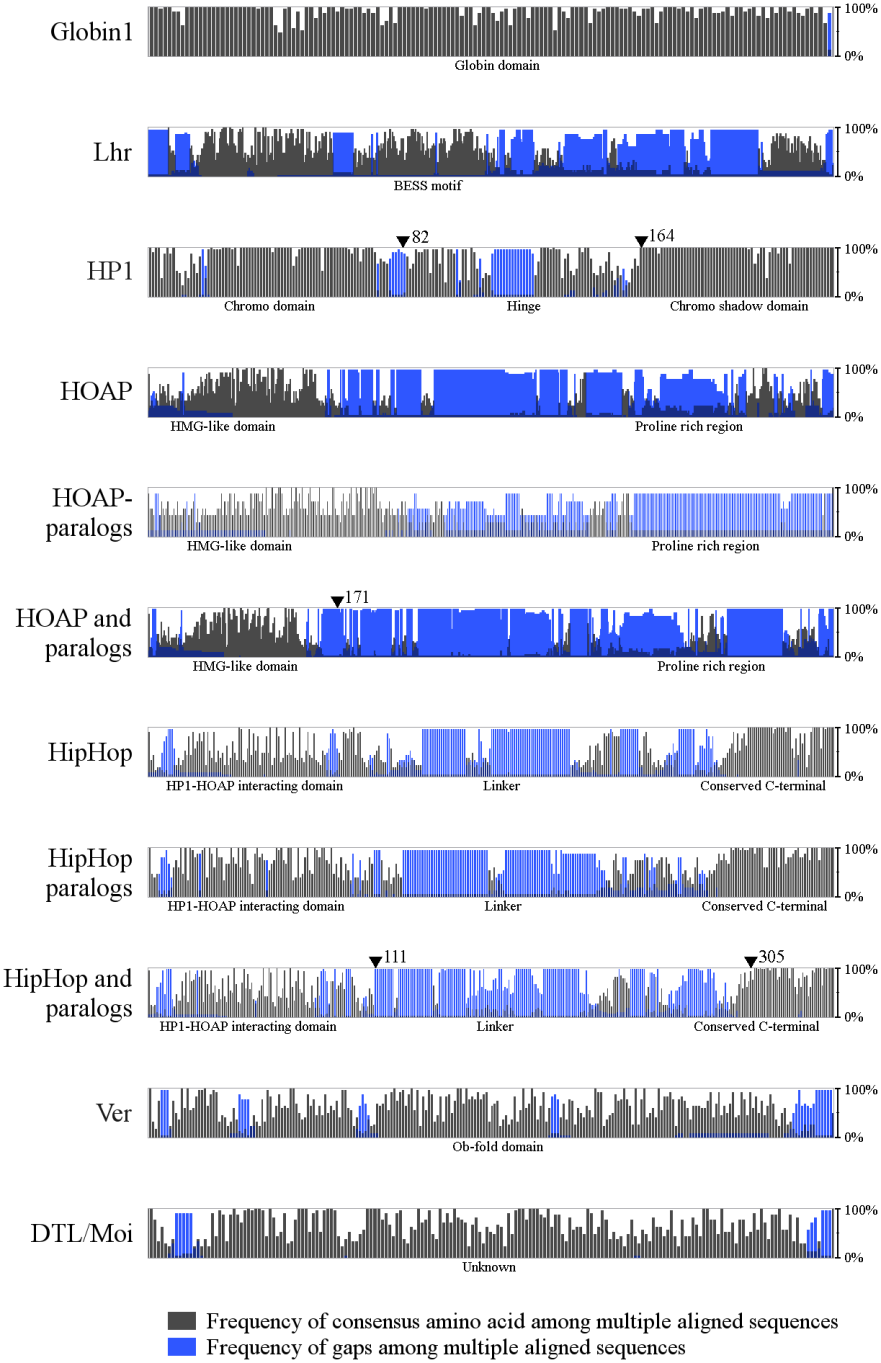
Sequence alignments reveal discrete parts of terminin proteins being subject to accelerated evolution. Homology plots show different conservation rates in distinct parts of terminin proteins. Each column represents the percentage of the consensus amino acid at the given position. Blue columns indicate regions where a gap occurs in some of the compared sequences as a result of deletion or insertion. Arrowheads and numbers represent domain boundaries considered during pN/pS calculations.

In order to determine the pN/pS values for protein domains we compared available terminin sequences from 21 *Drosophila* species. First the sequences were aligned to each other, then homology plots were calculated based on the alignments (Fig 1). We also included the conserved Globin1 [24] and the fast-evolving Lethal hybride rescue (Lhr) [25] proteins in our calculations as reference points.

Homology plots show the percentage of the most frequently found amino acid for each position in the compared sequences. We used these plots to determine whether the speed of evolution is uniform within molecules. We found that in most cases homology plots clearly showed that different parts of terminin protein molecules have evolved at different rates. Areas with similar amino acid conservation values correspond generally to protein domains identified earlier (Fig 1).

**Fig 2:**
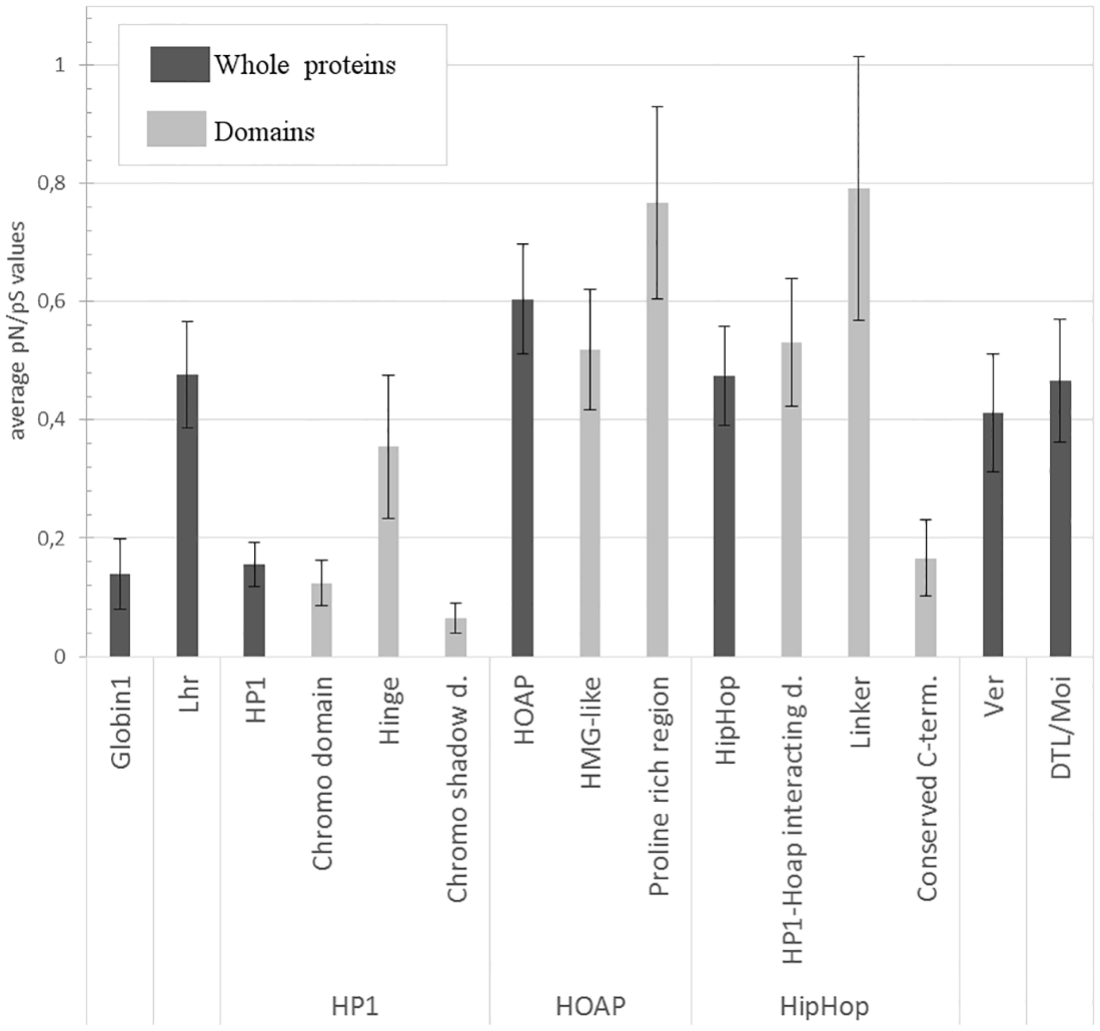
pN/pS values for entire proteins and for domains of terminin proteins indicate high rate of evolution. The pN/pS values shown in the diagram are averages of values obtained by pairwise comparisons with error bars representing standard deviation. Globin1 and Lhr represent conserved and fast-evolving proteins, respectively, as reference points.

We calculated the pN/pS ratio (based on codon alignments) for each identified domain (Fig 2) in order to compare the speed of evolution between and within molecules. The presented pN/pS values are averages of pairwise calculations. Since the evolution of the full-length proteins has already been studied [14, 15, 17, 18] we compared the evolution of domains to that of the whole proteins. Protein domains having pN/pS values below 0.2 according to our calculations were considered as conserved, whereas domains having pN/pS values above 0.4 were considered as fast-evolving ones. For comparison: similar calculations for the conserved globin1 and fast-evolving Lhr proteins of Drosophilae yielded values of 0.14 and 0.48, respectively. We found that the changes observed within terminin proteins follow characteristic patterns as summarized below.

HP1 [26, 27] is a conserved protein that has a low overall pN/pS ratio (0,16). The protein contains three domains, namely the chromo domain [28], the chromo shadow domain [29, 30] and a hinge region linking these two. We found the chromo and chromo shadow domains to be highly conserved (0.12 and 0.06 pN/pS ratio), whereas the hinge region shows higher variability (0.35) (Fig 1).

The HOAP protein can be divided into the HMG-like domain and the proline-rich region, which is responsible for interaction with HP1 hinge and chromo shadow regions [31]. According to the homology plot the HMG-like domain shows higher conservation values than the proline-rich region. The proline-rich region shows greater sequence diversity, which results in poor alignment with frequent gaps. However, a conserved motif could be identified at the C-terminal of this region (Fig 1). We also compared the 7 known paralogs of HOAP [32], and found that they show a similar pattern of conservation (Fig 1), therefore the paralogs were also included in pN/pS calculations. The results show that HOAP is a fast-evolving protein (0.60) and both of its domains have high pN/pS values (0.52 and 0.77) (Fig 2).

HipHop can be divided into three parts based on the speed of its evolution: it has a conserved C-terminal region, which is believed to be responsible for localizing HipHop in heterochromatin [33], a variable HP1-HOAP interacting domain [17], and an extreme variable region, which connects the two. These domains and their evolutionary speed have been studied and the findings are described [33]. The homology plots that we calculated for the 21 known HipHop amino acid sequences are in accord with the data available on HipHop evolution [33] (Fig 1). Similarly to HOAP, HipHop has many paralogs [32]. (The HipHop paralog in *D*. *melanogaster* is called K81, which is described as a “paternal effect gene” [34].) We found that the 15 HipHop paralogs displayed similar patterns in homology plots as HipHop (Fig 1). The results concerning the evolution of HipHop domains obtained from comparisons including 36 HipHop and paralog sequences (Fig 2) were consistent with the expectations, indicating that the C-terminal domain is highly conserved (0.165), whereas the other two domains show rapid rates of evolution (0.53 and 0.79).

Ver consists of an Ob-fold domain as described by Raffa et al. [18]. Ob-fold domains are responsible for oligosaccharide or oligonucleotide binding. Ver is supposed to bind single-stranded DNA [18]. The only known paralog of Ver (in *D*. *willistoni)* was also included in our calculations. Based on homology plots, regions of the Ver molecule show high conservation values; however, these regions are stretches not longer than 10 amino acids (Fig 1). Because the Ob-fold domain of Ver corresponds to nearly the entire protein, we calculated the pN/pS values for the whole sequence. The result confirmed the fast evolution of Ver (0,411) (Fig 2).

DTL/Moi has no identified domain structure. The homology plot did not reveal domain boundaries either; however, short conserved motifs can be found within the DTL/Moi sequences (Fig 1). This suggests that DTL/Moi is a single-domain protein, consequently we calculated pN/pS values for the whole sequences. The data show that DTL/Moi is a fast-evolving protein, as it was expected (0.47) (Fig 2).

Taken together, our analysis demonstrates that the pN/pS ratio calculated for a whole molecule could be misleading because of its statistical characteristics. A protein can consist of fast-evolving regions and conserved domains, and it depends on the ratio of these whether or not a whole protein can be considered to be fast–evolving. Conserved domains are usually functional parts of the molecules, whereas variable parts often serve as spacers or perhaps have a role in regulation. In the case of telomere capping proteins even the functional domains are variable as much as linker regions are in other molecules, which indicates that their evolution proceeds with a remarkable speed. Such fast-evolving domains with potential roles in protein-protein or protein-DNA interactions are the HMG-like domain of HOAP, the HP1-HOAP-interacting domain of HipHop, the Ob-fold domain of Ver and the DTL/Moi domain. These are ideal targets for studying the effect of accelerated evolution on complex assembly and can be exploited in planning *in vitro* experiments.

### Bacterial expression of terminin proteins

**Fig 3:**
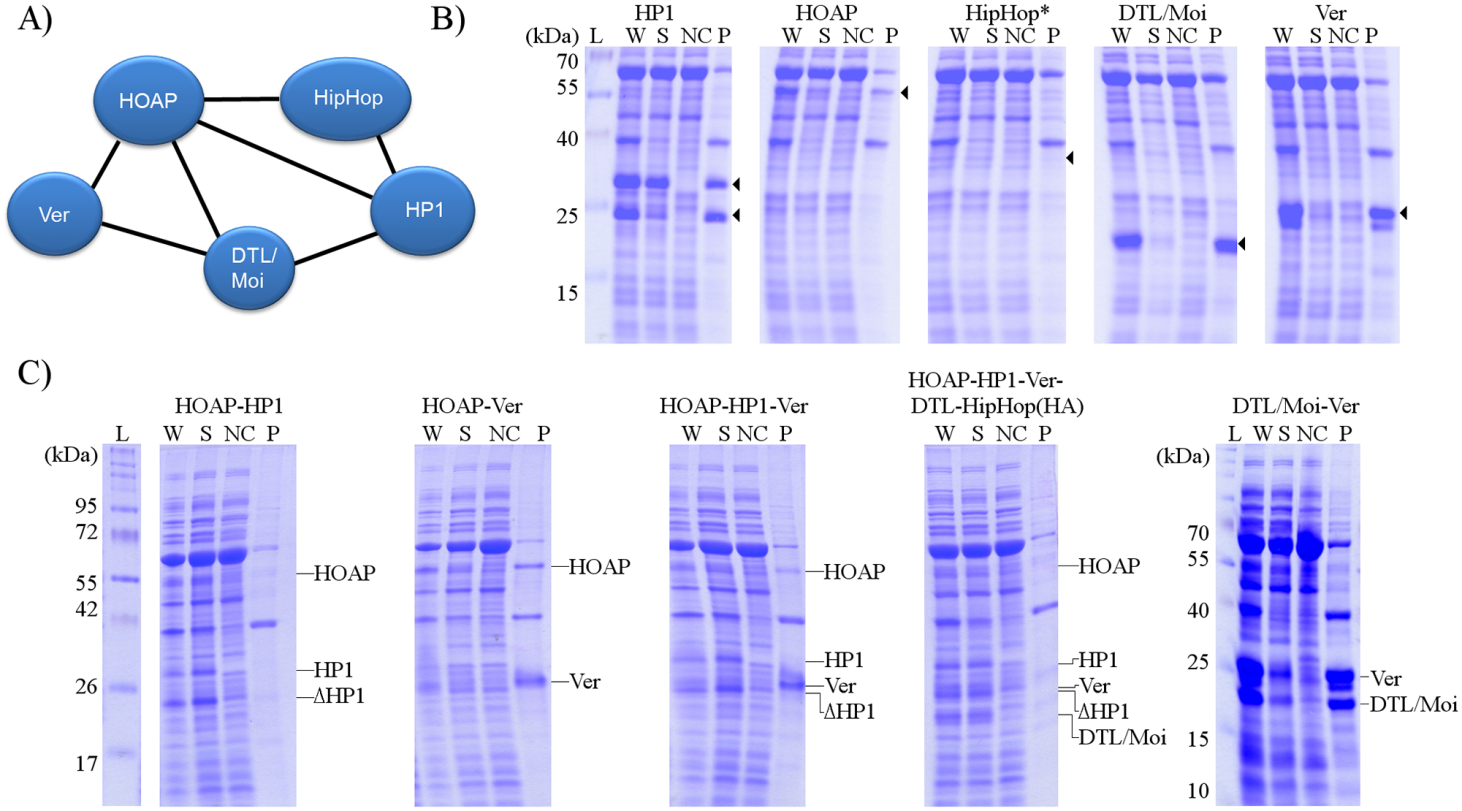
Co-expression of interacting partners increases the solubility of terminin proteins. (A) Presumed interactions among terminin proteins based on GST pull-down assays [17–19]. (B) Solubility of terminin components expressed individually in Arctic Express cells. Arrowheads point to bands corresponding to specific terminin proteins. HipHop expression cannot be observed on Coomassie-stained gel. On panel B images of different parts of the same gel are shown. (C) Co-expression of terminin components from polycistronic constructs. On panel C different parts of the same gel are shown except for the part with DTL/Moi-Ver data. L: molecular weight marker ladder, W: whole cell extract, S: supernatant, P: pellet, NC: control supernatant without any heterologous protein.

Previous studies have indicated interactions among terminin components as summarized in Fig 3A. In brief: Ver interacts with DTL/Moi and HOAP [18], and DTL/Moi interacts with Ver, HOAP and HP1 [19]. However, HipHop does not interact directly with Ver or DTL/Moi [17, 18, 19]. To verify these interactions and to explore more connections between specific terminin proteins, furthermore to uncover signs of co-evolution which might play a role in speciation, we studied heterologously expressed terminin proteins. Our strategy to obtain recombinant terminin components for complex assembly studies involved cloning cDNA sequences into expression vector(s) and producing the proteins in bacteria. For each of the five proteins we attempted to express, we used cDNA fragments encoding the complete coding regions, nonetheless in some cases we observed expression of shorter products resulting from degradation (see later in more detail). The use of monocistronic constructs revealed that HP1, HOAP, Ver and DTL/Moi were expressed at high level upon induction in BL21DE3 Codon Plus RIL cells. HP1 appeared in denaturing gels as two bands, the lower being an N-terminal truncation, which was present even if the cells were lysed directly in SDS sample buffer after harvesting. HipHop expression was consistently low, and despite various attempts which included alterations in construct designs, conditions of induction and choices of host cells and as well trials of co-expression with other terminin proteins, we could not achieve notable expression. The expression of HipHop at low level was, however, verified by immunodetection of the heterologously expressed HA tag-labelled protein [data not shown].

The heterologous expression of the majority of terminin proteins, however, resulted in an insoluble pellet after cell lysis, as judged by comparing Coomassie-stained samples on SDS-PAGE. Using Arctic Express cells as host, the solubility of HOAP, HP1 improved (by more than 50%), whereas the majority of Ver and DTL/Moi proteins remained in inclusion bodies.

Often the insolubility of expressed proteins can be overcome by co-expression of interacting partners [35, 36, 37]. In line with this logic, instead of purifying individual subunits for reconstitution attempts, we constructed polycistronic plasmids for simultaneous expression of various cDNAs. We found that co-expression of Ver with DTL/Moi and of HP1 with HOAP increased the solubility of these proteins quite differently: in the case of Ver and DTL/Moi it resulted in a barely detectable improvement on Coomassie-stained SDS-PAGE, whereas HOAP and HP1 co-expression resulted in nearly completely soluble proteins (Fig 3). Co-expression of Ver and HOAP or Ver, HOAP and HP1 did not increase the solubility of Ver. However, co-expression of all four proteins, namely HOAP, HP1, Ver and DTL/Moi resulted in soluble Ver and DTL/Moi proteins (Fig 3C). These observations indicate that soluble terminin proteins can be produced by their co-expression. These results also suggest that the presence of HipHop is not an absolute requirement for complex formation. This notion is in accord with the presumed interactions among these molecules based on earlier studies [17–19] (Fig 3A), which suggests that a stable heterotetramer terminin subcomplex may form in the absence of HipHop.

### Purification of Ver and DTL/Moi

**Fig 4:**
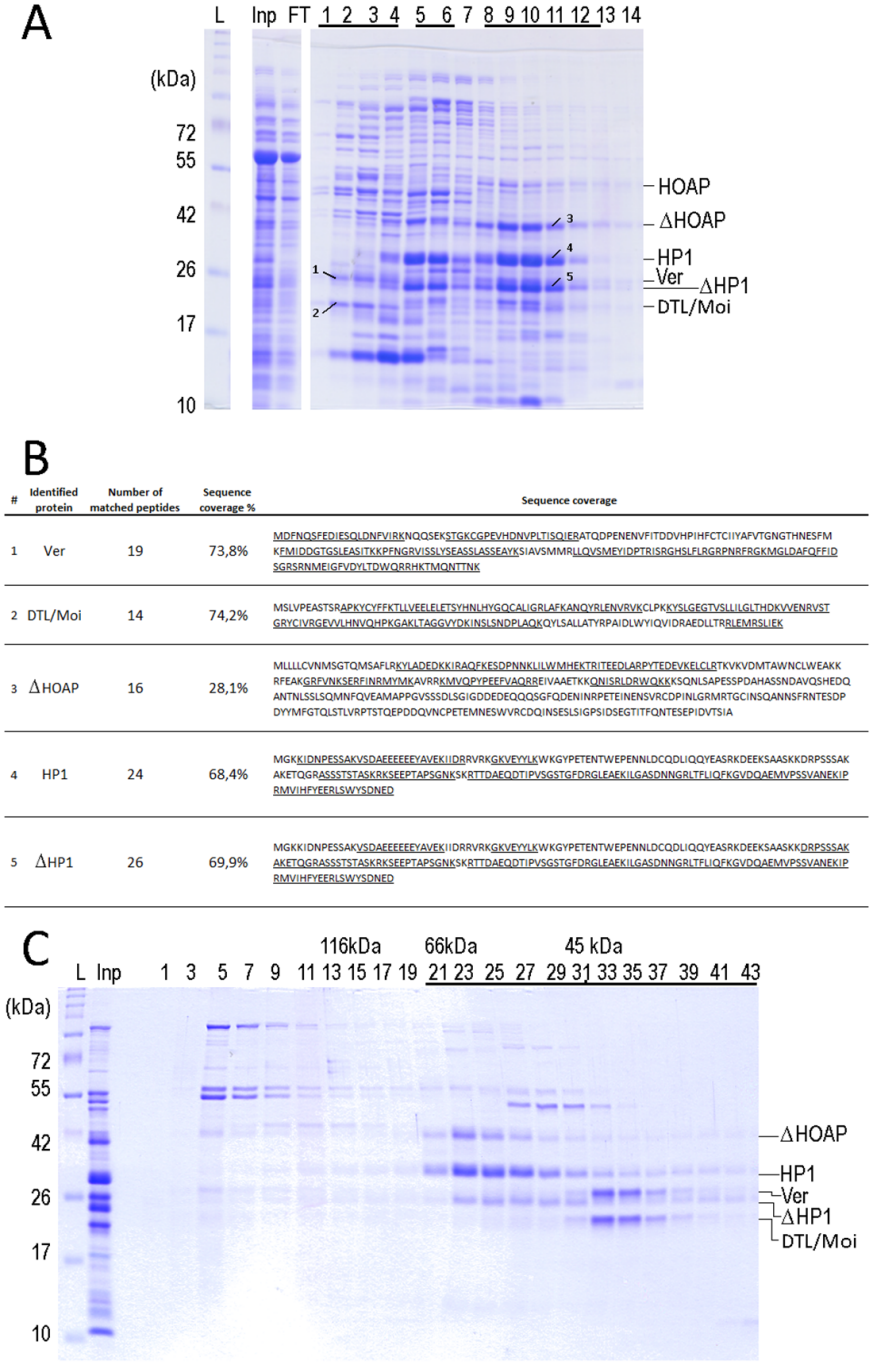
Terminin components co-purify as discrete sub-complexes. (A) PAGE of protein fractions eluted by increasing salt gradient from a heparin-sepharose column onto which a cell lysate containing co-expressed HOAP, Ver, DTL/Moi and HP1 proteins was loaded. The tricine SDS-gel was stained by Coomassie. Ver and DTL/Moi co-eluted in the first 4 fractions, whereas HOAP and HP1 co-eluted at higher salt concentrations. HP1 elutes in two peaks: in fractions 4–6, and fractions 8–12, the latter being observed only if HOAP is present. Protein identities were confirmed by either western blot or mass spectrometry (Panel B). On panel A different parts of the same gel are shown. The bands marked were subjected to mass spectrometry. (B) Results of mass spectrometry identification of heterologously expressed terminin proteins. The peptide regions identified by mass spectrometry are underlined in the amino acid sequences of expressed proteins. (C) Fractions of gel filtrations obtained after heparin-sepharose column purification: fractions containing HOAP with HP1 and Ver with DTL/Moi were re-mixed at low salt concentration and gel filtrated. Molecular weight marker ladder (L), input (Inp), flow-through (FT) and fraction numbers and the position of the respected proteins are indicated.

In order to gain insight into the subunit composition of the putative terminin complex, we subjected the lysate of cells that co-expressed the four heterologous proteins (HOAP, HP1, Ver and DTL/Moi) to chromatography on heparin-sepharose column. (The weak ion exchange matrix was chosen because of its proven suitability for purification of DNA-binding proteins). We found that Ver together with DTL/Moi, and similarly HOAP together with HP1 eluted in different fractions (Fig 4A). Although full-length proteins were expressed, the HOAP proline-rich region was truncated during purification. Supplementing the four co-expressed proteins with samples of HipHop obtained from a larger volume did not change the above-described result: not surprisingly HipHop co-eluted with HOAP and HP1.

We subjected peak fractions from the heparin-sepharose matrix to gel filtration at low salt concentration to investigate the existence of two sub-complexes (Fig 4C). In the case of co-eluted HOAP and HP1 the stoichiometry remained unclear, since a shorter form of HOAP (~40 kDA) was also present in the samples and co-purified with the full-length HOAP and HP1 proteins. According to mass spectrometry, this represented a truncated form of HOAP that had lost part of its proline-rich region (Fig 4B). During gel filtration HOAP and HP1 co-migrate forming a broad peak that suggests the presence of several complex types. The sizes of complexes are between 66 and 40 kDa, suggesting that HP1 homodimers and dimers of HOAP and HP1 involving both truncated and full-length versions could be formed. Although in our experiments HOAP co-purified with HP1, indicating an interaction between the two proteins, we could not verify the 1:2 stoichiometry of HOAP:HP1 interaction reported by Badugu et al. [31]. This could be explained with the partial loss of the proline-rich region of HOAP. This region is supposedly responsible for interaction with the HP1 dimer [31]. Thus our observations suggest that secondary interacting surfaces may be present.

**Fig 5:**
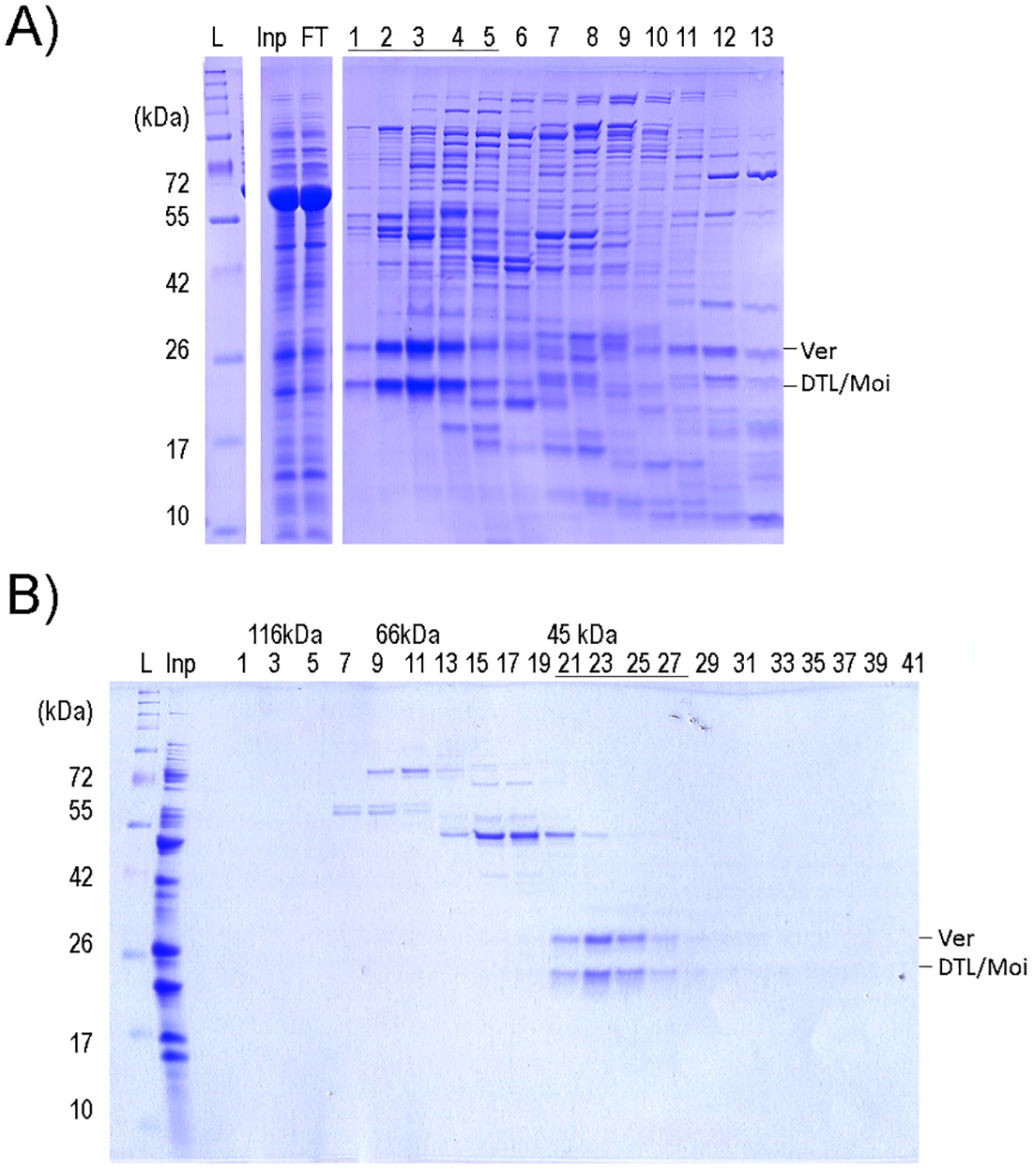
Ver and DTL/Moi form a heterodimer that could be purified independently from other terminin proteins. (A) Heparin-sepharose chromatography fractions of co-expressed Ver and DTL/Moi. Different parts of the same gel are shown. (B) The first 5 fractions of the purification shown on panel A containing Ver and DTL/Moi proteins were combined and gel-filtrated on Superdex 200 10/300 GL column. Molecular weight marker (L), input (Inp), flow through (FT) and fraction numbers and the position of the respected proteins are indicated.

Mixing the peak fractions that eluted from the heparin-sepharose matrix at low salt did not change the profile of the subsequent gel filtration, indicating that the elution of Ver-DTL/Moi and HOAP-HP1 in two peaks from heparin-sepharose is not due to the increasing salt concentration used during development of the column (Fig 4C). The formation of a stable heterodimer of Ver and DTL/Moi was also verified by processing the soluble fraction from bicistronic expression similarly as described above: Ver and DTL/Moi were bound to a heparin-sepharose column and eluted at low NaCl concentration (Fig 5A). During gel filtration Ver and DTL/Moi proteins co-migrated as one single peak corresponding to a 45 kDa mass, which contained the two proteins in 1:1 ratio as expected (Fig 5B).

**Fig 6:**
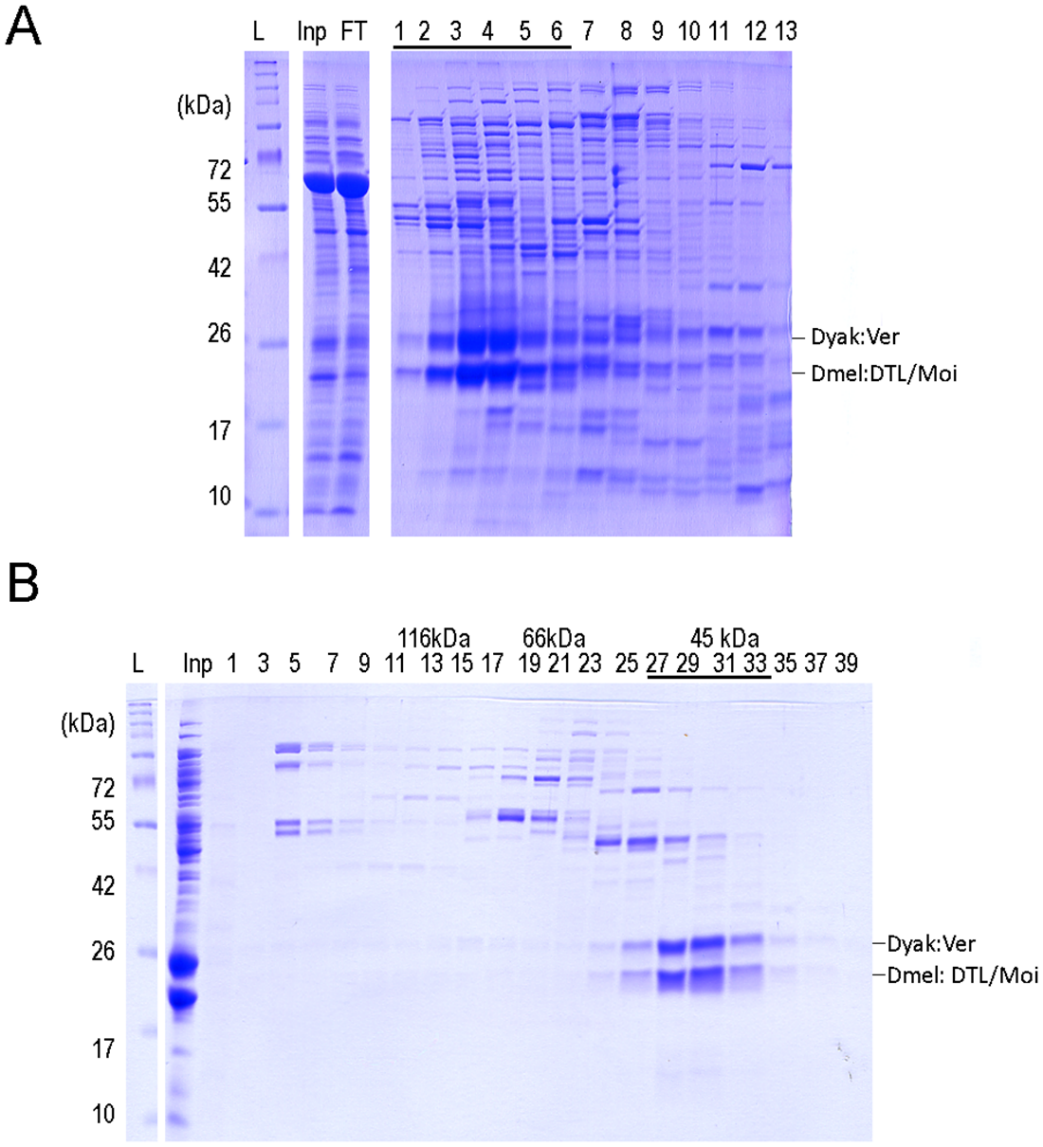
*D*. *yakuba* Ver and *D*. *melanogaster* DTL/Moi form co-purifying hybrid complex. (A) Cell lysate containing co-expressed *D*. *yakuba* Ver and *D*. *melanogaster* DTL/Moi was loaded onto heparin-sepharose column and the proteins were eluted by an increasing NaCl gradient. The protein content of the fractions was analysed on tricine SDS-PAGE. Different parts of the same gel are shown. (B) Fractions of the purification shown above containing *D*. *yakuba* Ver and *D*. *melanogaster* DTL/Moi (fractions:1–6) were combined and gel-filtrated on Superdex 200 10/300 GL column. The two proteins eluted in the same fractions in 1:1 ratio at 45kDa suggesting the formation of a hybrid complex. Molecular weight marker (L), input (Inp), flow through (FT) and fraction numbers, and the position of the respected proteins are indicated.

As noted above, DTL/Moi and Ver are both rapidly evolving proteins. *Drosophila yakuba* Ver is 83% identical with *D*. *melanogaster* Ver. The identity of *D*. *melanogaster* DTL/Moi and *D*. *yakuba* DTL/Moi is 89%. (The identity between Globin1 and Lhr proteins of these two Drosophila species is 98% and 69%, respectively.) One can assume that the differences between these proteins could influence protein-protein interactions and may affect protein function as well, therefore might have contributed to the isolation of species. To attempt an experimental verification of this concept we investigated if *D*. *yakuba* Ver could form a complex with *D*. *melanogaster* DTL/Moi. For this we co-expressed and purified the two proteins. We found that they are able to bind to heparin-sepharose column as a hybrid complex and can be eluted similarly to the dimer of the two corresponding *D*. *melanogaster* proteins (Fig 6A). The formation of the *D*. *yakuba* Ver and *D*. *melanogaster* DTL/Moi dimer was successfully demonstrated by gel filtration as well (Fig 6B). Thus, we concluded that *D*. *yakuba* Ver forms a stable heterodimer with *D*. *melanogaster* DTL/Moi.

## Discussion

We attempted to reconstitute the *Drosophila* terminin complex from proposed terminin proteins expressed in bacteria either individually or together from polycistronic transcription units. Our efforts to express HipHop in larger amounts failed repeatedly, hampering our attempts. Though our system is limited in this respect, existing data on pairwise interactions between terminin proteins suggest that the presence of HipHop might not be an absolute requirement for complex formation, and a heterotetramer terminin subcomplex may form in the absence of HipHop. Remarkably, we did not observe the presence of a tetrameric complex during purification of four co-expressed capping proteins, but detected subcomplexes consisting of Ver-DTL/Moi and HOAP-HP1. Moreover, the Ver-DTL/Moi and HOAP-HP1 sub-complexes were eluted in different fractions. This might indicate that the described protein interactions are not equally important to hold a multisubunit complex together. In particular the HOAP-DTL/Moi, HP1-DTL/Moi and HOAP-Ver interactions seem to be weaker compared to interactions forming between the previous two pairs of proteins. On the other hand, we cannot exclude the possibility that HipHop might be an essential component for complex stability. HOAP is needed for the localization of Ver and DTL/Moi at the telomeres, and interactions were detected among these proteins by GST-pull-down assays [18, 19]. In experiments performed on artificial telomeres HOAP, HipHop and HP1 co-localized, whereas DTL/Moi did not [17]. Our data are in accord with results of these studies. In summary, despite limitations of the heterologous expression systems, such as lack of posttranslational modifications and probing terminin complex formation in the lack of specific DNA structure, we have successfully produced two subcomplexes of terminin proteins. The subsequent steps of complex assembly might require the presence of DNA or involve non-terminin proteins that have a role in telomere maintenance.

The fast evolution of capping proteins raises several interesting questions regarding co-evolution of interacting proteins and the possible role of terminin formation in speciation.

Proteins that have a crucial function in telomere maintenance are expected to be conserved to preserve function, yet terminin proteins show accelerated evolution. This contradiction could be resolved by two reasonings. The first option is that, although these molecules are considered to be fast-evolving proteins—based on their pN/pS ratio or similarity data—they actually contain conserved domains that are responsible for their conserved function. An example for this could be the conserved C-terminal of HipHop that is a functioning part of the molecule. Another solution could be that the functional domains also evolve rapidly. In that case the domains must be able to accumulate many mutations without affecting the main function of the proteins. We found four domains, the HMG-like domain of HOAP, the HP1-HOAP interacting domain of HipHop, the Ob-fold domain of Ver and the uncharacterized DTL/Moi domain that show accelerated evolution and still maintain function. In these domains there are only short—a few amino acid long—conserved motifs. We hypothesize that these motifs might be positioned next to each other during folding to create a core which ensures a proper structure that is responsible for the function of the protein. Several amino acid variations could serve to position these motifs correctly, therefore the lower selection pressure explains the fast evolution. Members of the Ob fold protein family can serve as good examples to demonstrate that an identical structure could be produced by different sequences [38]. Other studies have also reported on fast-evolving proteins with conserved function [39, 40].

We hoped that examination of the interaction of DTL/Moi and Ver can provide information on whether their accelerated evolution has a role in forming species barriers. We reasoned that a lack of interaction between terminin proteins might be used as an argument for a role in speciation. Because we wished to capture speciation in progress, we selected Drosophila species, which are in close evolutionary relationship. We reasoned that more distant species are more likely to develop other speciation barriers, which could be based on differences in morphology, behavior, size, etc. We found that *D*. *yakuba* Ver, which differs in 17% of its amino acids from *D*. *melanogaster* Ver forms a complex with *D*. *melanogaster* DTL/Moi in spite of the 11% difference between *D*. *melanogaster* DTL/Moi and *D*. *yakuba* DTL/Moi. Thus the observed existence of interspecies interaction might be interpreted as a counter argument against a role of the terminin proteins in speciation. However, like in other cases, proving a “no role” is difficult, since several factors, among them the degree of divergence, the species included, the roles of the tested proteins in the complex and several others should be considered. The complexity of the problem is demonstrated by a recent publication of Fukagawa [41], which explains the mechanism by which *Lethal hybrid rescue* (*Lhr)* and *Hybrid male rescue (Hmr)* act as hybrid incompatibility genes. Interestingly, the *D*. *simulans* Lhr protein is unable to cause male lethality if the *D*. *melanogaster hmr* gene product is not present in the hybrid, therefore an interaction between these proteins is required in order to function as speciation barrier [42]. This observation suggests that it is the altered function and not the loss of function of the hybrid complex that has a role in separating species. The cross-species stable heterodimer of *D*. *yakuba* Ver and *D*. *melanogaster* DTL/Moi might need to be considered similarly.

Therefore the hypothesis that terminin proteins play role as speciation barrier remains to be tested. To examine this question more thoroughly, experiments are needed to test functional changes in hybrid complexes both *in vitro* and *in vivo*. Our experimental system of producing the terminin proteins and the results of our analysis of identifying their fast-evolving regions provide a base to plan these experiments.

## Materials and Methods

### Sequence alignments

The *Drosophila melanogaster* genome regions corresponding to terminin genes were downloaded from Flybase. These sequences were used for Blastn searches in whole-genome shotgun contig databases of 21*Drosophila* species (*Drosophila ananassae*, *D*. *biarmipes*, *D*. *bipectinata*, *D*. *elegans*, *D*. *erecta*, *D*. *eugracilis*, *D*. *ficusphila*, *D*. *grimshawi*, *D*. *kikkawai*, *D*. *melanogaster*, *D*. *miranda*, *D*. *mojavensis*, *D*. *persimilis*, *D*. *pseudoobscura pseudoobscura*, *D*. *rhopaloa*, *D*. *sechellia*, *D*. *simulans*, *D*. *takahashii*, *D*. *virilis*, *D*. *willistoni*, *D*. *yakuba*). The *Drosophila albomicans* sequences were not used in this study because of the preliminary stage of sequence data processing at the time of the analysis we performed. The initial blast searches did not result hits in each species therefore results of first searches were used as quarries for further Blastn searches to detect missing sequences. Dubruille et al. have described many HipHop and HOAP orthologs and paralogs in detail [32] we complemented our sequence identification data with their findings. We have identified further HOAP and HipHop orthologs in *Drosophila erecta*, *D*. *miranda*, *D*. *sechellia*, and *D*. *yakuba*. We have also identified a HOAP duplication in *D*. *miranda*, based on sequence similarity. The paralog and ortholog sequences were distinquished by their genomic environment. We have downloaded the extended sequence of the blast hits and predicted the coding regions by Augustus software (http://bioinf.uni-greifswald.de/augustus) [43]. In those cases when Augustus was unable to detect any open reading frame (some Verrocchio sequences) the coding region was determined by Ugene software (Unipro) [44]. The locations of the coding regions and the annotation numbers (GI) of the sequences used are described in S1 Table. The predicted coding sequences were translated into amino acid sequences and aligned by T-coffee algorithm with standard settings (-50 gap opening penalty) in Ugene. Homology plots were also calculated. The codon alignments were based on these amino acid alignments and generated by PAL2NAL [45] software. The ratio of non-synonymous and synonymous substitutions in the codon alignments were calculated by SNAP for each of sequence pair (www.hiv.lanl.gov) [46].

### Cloning

Full-length cDNAs were obtained from *Drosophila* Genomics Resource Center, Indiana University, Bloomington. In order to construct expression plasmids cDNAs were amplified in high fidelity PCR reactions using Phusion (Thermo Scientific) polymerase using primers listed below:

HP1: GACACCATGGGCAAGAAAATCGACAACCCTGAGAGCTC, GACAGGATCCTTAATCTTCATTATCAGAGTAC;
HOAP: GACACCATGGCACTGCTGCTACTATGTGTTAATATGTCGGGGAC, GACAGGATCCTCAGGCTATTGAGGTGACGTC;
HipHop: GACACATATGGCCTCCATTGACGAGGGCTCGCGCGTTGAGCGGAG, GACAGGATCCCTAACCACCTGTGGTTCCCATC;
DTL/Moi: GTACCATGGTTATGTCCCTGGTGCCAGAAGCCT, GTAGGATCCTCATTTCTCGATCAGACTTCTCATCTCCA;
Ver: GTACATATGGATTTTAATCAGAGTTTCGAGG, CAAAGATCTCTATTTATTTGTTGTATTCTGCATTG.

In order to construct polycistronic expression vectors, inserts were amplified using monocistronic expression plasmids as templates with the help of the following primer pair: (CCCTCTAGAAATAATTTTGTTTAACTTTAAGAAGGAGATATA, ATAGATCTGCGGCCGCACTAGTAACTCAGCTTCCTTTCGGGCTTTGTTAG).

The forward primer hybridizes with the ribosome binding site of the pET expression vector and the reverse primer binds the sequence before the start of the T7 transcription termination signal. The resulting products have the following structure: *XbaI*–ribosome binding site–cDNA–*SpeI*–*NotI*–*BglII*. *pET22b* vector plasmid and the insert were digested by *XbaI* and *NotI* and ligated to construct a monocistronic expression plasmid. Then, this was digested with *SpeI* and *NotI*, while the next insert was digested with *XbaI* and *NotI* enzymes. Note, that *XbaI* and *SpeI* restriction endonucleases produce compatible ends. The steps resulting in a bicistronic construct can be reiterated as in every subsequent ligation step the *SpeI* site on the plasmid is eliminated while the *NotI* site remains available for cloning and a new *SpeI* site is introduced with the insert.

### Protein expression and Tricine SDS-PAGE

Protein production was performed in Arctic express cells, DE3 (Agilent Technologies, Inc.) at 18°C. Induction was done with 0.3 mM IPTG for 48–60 hours. Cells were lysed by sonication using Sonics Vibra cell^™^ apparate. Each sample was sonicated for 6 cycles of 20 second active sonication with 10 sec breaks at 30% amplitude in sonication buffer (25 mM Tris HCl pH7.5, 100 mM NaCl, 1mM CaCl_2_, 1 mM MgCl_2_).

Proteins were separated on 10% Tricine-SDS-PAGE (gel buffer (1M Tris, 0.33M HCl, 0,1% SDS pH 8.45), anode buffer (0.1M Tris, 0.022M HCl, pH 8.9), cathode buffer 10x (0.1M Tris, 0.1M Tricine, 0.1% SDS, pH8,25) and visualized by Coomassie Brilliant Blue staining as described [47].

### Chromatography

Cell extracts were cleared by centrifugation and filtration and loaded to heparin-sepharose column (GE Healthcare) at 1ml/min flow rate. Proteins were eluted by a 0.1 to 1M NaCl gradient in 20 mM Tris pH7.5 in 20 column volume and 1 ml fractions were collected.

Gel filtration was performed on Superdex 200 10/300 GL column (GE Healthcare) using filter concentrated fractions from heparin-sepharose purification. 20 mM Tris pH7.5 100mM NaCl was used with 0.25ml/min flow rate, 0.3ml fractions were collected. The column was calibrated using the Broad range SDS-PAGE Standard (BioRad). The peaks of the 116, 66 and 45 kDa proteins were marked on the related figures (Figs 4C, 5B and 6B).

### Peptide mass fingerprinting

Bands corresponding to specific proteins separated on Tricine SDS-PAGE and stained by Coomassie Blue were cut and after reduction and alkylation the proteins were digested by trypsin in the gel following the protocol described [48]. The trypsin activity was inhibited by addition of 10% formic acid. Samples were extracted from the gel by sonication and were desalted using C18 resin. Then samples were mixed with dihydroxybenzoic acid in 1:1 ratio and loaded to the MALDI target plate. The plate was dried on room temperature. After calibration the sample were analyzed on MALDI-ToF using ‘RP_2-3kDa-med’ parameters. Spectrum was taken and monoisotopic peaks were selected by FlexAnalysis software. Proteins were identified by MASCOT and Protein Prospector search in SwissProt.2014.3.7 database.

## Supporting Information

S1 Table The Gi numbers or annotation symbols of sequences that used in this study. *In Drosophila melanogaster DTL/Moi is translated from the same transcription unit as the adjacent Tgs1 gene [49]. The structure of transcriptions unit(s) that specifies DTL/Moi and Tgs1 proteins varies among Drosophila species. In order to clarify the margins of analyzed sequences further general database reference was added to annotation symbols. Green background represents HOAP orthologs and paralogs while blue background represents HipHop orthologs and paralogs mentioned by Dubruille et al. [32].
(XLSX)

## References

[1] Olovnikov AM. Principle of marginotomy in template synthesis of polynucleotides. Dokl Akad Nauk SSSR. 1971;201(6):1496–9. 5158754

[2] Watson JD. Origin of concatemeric T7 DNA. Nat New Biol. 1972 10 18;239(94):197–201. 4507727 10.1038/newbio239197a0

[3] Lydall D. Taming the tiger by the tail: modulation of DNA damage responses by telomeres. EMBO J. 2009 8 5;28(15):2174–87. 10.1038/emboj.2009.176 Epub 2009 Jul 23. 19629039 PMC2722249

[4] Greider CW, Blackburn EH. Identification of a specific telomere terminal transferase activity in Tetrahymena extracts. Cell. 1985 12;43(2 Pt 1):405–13. 3907856 10.1016/0092-8674(85)90170-9

[5] Griffith JD, Comeau L, Rosenfield S, Stansel RM, Bianchi A, Moss H, et al Mammalian telomeres end in a large duplex loop. Cell. 1999 5 14;97(4):503–14 10338214 10.1016/s0092-8674(00)80760-6

[6] Palm W, de Lange T. How shelterin protects mammalian telomeres. Annu Rev Genet. 2008;42:301–34. 10.1146/annurev.genet.41.110306.130350 18680434

[7] Fulcher N, Derboven E, Valuchova S, Riha K. If the cap fits, wear it: an overview of telomeric structures over evolution. Cell Mol Life Sci. 2014 3;71(5):847–65. 10.1007/s00018-013-1469-z Epub 2013 Sep 17. 24042202 PMC11113737

[8] Louis EJ. Are Drosophila telomeres an exception or the rule? Genome Biol. 2002 9 27;3(10):REVIEWS0007 Epub 2002 Sep 27. 12372147 10.1186/gb-2002-3-10-reviews0007 PMC244910

[9] Cenci G, Ciapponi L, Gatti M. The mechanism of telomere protection: a comparison between Drosophila and humans. Chromosoma. 2005 8;114(3):135–45. Epub 2005 Jul 13. 16012858 10.1007/s00412-005-0005-9

[10] Mason JM, Frydrychova RC, Biessmann H. Drosophila telomeres: an exception providing new insights. Bioessays. 2008 1;30(1):25–37. 18081009 10.1002/bies.20688 PMC2804870

[11] Mason JM, Biessmann H. The unusual telomeres of Drosophila. Trends Genet. 1995 2;11(2):58–62. 7716808 10.1016/s0168-9525(00)88998-2

[12] Pardue ML, DeBaryshe PG. Retrotransposons provide an evolutionarily robust non-telomerase mechanism to maintain telomeres. Annu Rev Genet. 2003;37:485–511. 14616071 10.1146/annurev.genet.38.072902.093115

[13] Villasante A, de Pablos B, Méndez-Lago M, Abad JP. Telomere maintenance in Drosophila: rapid transposon evolution at chromosome ends. Cell Cycle. 2008 7 15;7(14):2134–8. Epub 2008 May 12. 18635962 10.4161/cc.7.14.6275

[14] Raffa GD, Ciapponi L, Cenci G, Gatti M. Terminin: a protein complex that mediates epigenetic maintenance of Drosophila telomeres. Nucleus. 2011 Sep-Oct;2(5):383–91. 10.4161/nucl.2.5.17873 Epub 2011 Sep 1. 21989238

[15] Shareef MM, King C, Damaj M, Badagu R, Huang DW, Kellum R. Drosophila heterochromatin protein 1 (HP1)/origin recognition complex (ORC) protein is associated with HP1 and ORC and functions in heterochromatin-induced silencing. Mol Biol Cell. 2001 6;12(6):1671–85. 11408576 10.1091/mbc.12.6.1671 PMC37332

[16] Cenci G, Siriaco G, Raffa GD, Kellum R, Gatti M. The Drosophila HOAP protein is required for telomere capping. Nat Cell Biol. 2003 1;5(1):82–4. 12510197 10.1038/ncb902

[17] Gao G, Walser JC, Beaucher ML, Morciano P, Wesolowska N, Chen J, et al HipHop interacts with HOAP and HP1 to protect Drosophila telomeres in a sequence-independent manner. EMBO J. 2010 2 17;29(4):819–29. 10.1038/emboj.2009.394 Epub 2010 Jan 7. 20057353 PMC2829166

[18] Raffa GD, Raimondo D, Sorino C, Cugusi S, Cenci G, Cacchione S, et al Verrocchio, a Drosophila OB fold-containing protein, is a component of the terminin telomere-capping complex. Genes Dev. 2010 8 1;24(15):1596–601. 10.1101/gad.574810 20679394 PMC2912556

[19] Raffa GD, Siriaco G, Cugusi S, Ciapponi L, Cenci G, Wojcik E, et al The Drosophila modigliani (DTL) gene encodes a HOAP-interacting protein required for telomere protection. Proc Natl Acad Sci U S A. 2009 2 17;106(7):2271–6. 10.1073/pnas.0812702106 Epub 2009 Jan 30. 19181850 PMC2650146

[20] Komonyi O, Schauer T, Papai G, Deak P, Boros IM. A product of the bicistronic Drosophila melanogaster gene CG31241, which also encodes a trimethylguanosine synthase, plays a role in telomere protection. J Cell Sci. 2009 3 15;122(Pt 6):769–74. 10.1242/jcs.035097 Epub 2009 Feb 24. 19240120

[21] Fanti L, Giovinazzo G, Berloco M, Pimpinelli S. The heterochromatin protein 1 prevents telomere fusions in Drosophila. Mol Cell. 1998 11;2(5):527–38. 9844626 10.1016/s1097-2765(00)80152-5

[22] Savitsky M, Kravchuk O, Melnikova L, Georgiev P. Heterochromatin protein 1 is involved in control of telomere elongation in Drosophila melanogaster. Mol Cell Biol. 2002 5;22(9):3204–18. 11940677 10.1128/MCB.22.9.3204-3218.2002 PMC133762

[23] Perrini B, Piacentini L, Fanti L, Altieri F, Chichiarelli S, Berloco M, et al HP1 controls telomere capping, telomere elongation, and telomere silencing by two different mechanisms in Drosophila. Mol Cell. 2004 8 13;15(3):467–76. 15304225 10.1016/j.molcel.2004.06.036

[24] Hankeln T, Jaenicke V, Kiger L, Dewilde S, Ungerechts G, Schmidt M, et al Characterization of Drosophila hemoglobin. Evidence for hemoglobin-mediated respiration in insects. J Biol Chem. 2002 8 9;277(32):29012–7. Epub 2002 Jun 4. 12048208 10.1074/jbc.M204009200

[25] Satyaki PR, Cuykendall TN, Wei KH, Brideau NJ, Kwak H, Aruna S, et al The Hmr and Lhr hybrid incompatibility genes suppress a broad range of heterochromatic repeats. PLoS Genet. 2014 3 20;10(3):e1004240 10.1371/journal.pgen.1004240 eCollection 2014. 24651406 PMC3961192

[26] James TC, Elgin SC. Identification of a nonhistone chromosomal protein associated with heterochromatin in Drosophila melanogaster and its gene. Mol Cell Biol. 1986;6:3862–3872. 3099166 10.1128/mcb.6.11.3862-3872.1986 PMC367149

[27] Eissenberg JC, James TC, Foster-Hartnett DM, Hartnett T, Ngan V, Elgin SC. Mutation in a heterochromatin-specific chromosomal protein is associated with suppression of position-effect variegation in Drosophila melanogaster. Proc Natl Acad Sci U S A. 1990 12; 87(24):9923–7. 2124708 10.1073/pnas.87.24.9923 PMC55286

[28] James TC, Gaunt SJ. A sequence motif found in a Drosophila heterochromatin protein is conserved in animals and plants. Nucl. Acids Res. 19, 789–794. 1708124 10.1093/nar/19.4.789 PMC333712

[29] Aasland R, Stewart AF. The chromo shadow domain, a second chromo domain in heterochromatin-binding protein 1, HP1. Nucleic Acids Res. 1995 8 25; 23(16): 3168–3173. 7667093 10.1093/nar/23.16.3168 PMC307174

[30] Koonin EV, Zhou S, Lucchesi JC. The chromo superfamily: new members, duplication of the chromo domain and possible role in delivering transcription regulators to chromatin. Nucleic Acids Res. 1995 11 11; 23(21):4229–33. 7501439 10.1093/nar/23.21.4229 PMC307373

[31] Badugu R, Shareef MM, Kellum R. Novel Drosophila heterochromatin protein 1 (HP1)/origin recognition complex-associated protein (HOAP) repeat motif in HP1/HOAP interactions and chromocenter associations. J Biol Chem. 2003 9 5;278(36):34491–8. Epub 2003 Jun 25. 12826664 10.1074/jbc.M305262200

[32] Dubruille R, Marais GA, Loppin B. Repeated evolution of testis-specific new genes: the case of telomere-capping genes in Drosophila. Int J Evol Biol. 2012;2012:708980 10.1155/2012/708980 Epub 2012 Jul 11. 22844639 PMC3401529

[33] Gao G, Cheng Y, Wesolowska N, Rong YS. Paternal imprint essential for the inheritance of telomere identity in Drosophila. Proc Natl Acad Sci U S A. 2011 3 10.1073/pnas.1016792108 PMC3064339 21383184

[34] Yasuda GK, Schubiger G, Wakimoto BT. Genetic characterization of ms (3) K81, a paternal effect gene of Drosophila melanogaster. Genetics. 1995 5;140(1):219–29. 7635287 10.1093/genetics/140.1.219 PMC1206549

[35] Henricksen LA, Umbricht CB, Wold MS. Recombinant replication protein A: expression, complex formation, and functional characterization. J Biol Chem. 1994 4 15;269(15):11121–32. 8157639

[36] Yao N, Coryell L, Zhang D, Georgescu RE, Finkelstein J, Coman MM, et al Replication factor C clamp loader subunit arrangement within the circular pentamer and its attachment points to proliferating cell nuclear antigen. J Biol Chem. 2003 12 12;278(50):50744–53. Epub 2003 Oct 6. 14530260 10.1074/jbc.M309206200

[37] Diebold ML, Fribourg S, Koch M, Metzger T, Romier C. Deciphering correct strategies for multiprotein complex assembly by co-expression: application to complexes as large as the histone octamer. J Struct Biol. 2011 8;175(2):178–88. 10.1016/j.jsb.2011.02.001 Epub 2011 Feb 12. 21320604

[38] Guardino KM, Sheftic SR, Slattery RE, Alexandrescu AT. Relative Stabilities of Conserved and Non-Conserved Structures in the OB-Fold Superfamily Int J Mol Sci. 2009 5; 10(5): 2412–2430. Published online 2009 May 22. 10.3390/ijms10052412 19564956 PMC2695284

[39] Brideau NJ, Barbash DA. Functional conservation of the Drosophila hybrid incompatibility gene Lhr. BMC Evol Biol. 2011 3 2;11:57 10.1186/1471-2148-11-57 21366928 PMC3060119

[40] Reinhardt JA, Jones CD. Two Rapidly Evolving Genes Contribute to Male Fitness in Drosophila. J Mol Evol (2013) 77:246–259 10.1007/s00239-013-9594-8 24221639 PMC3880551

[41] Fukagawa T. Speciation mediated by centromeres. Dev Cell. 2013 11 25;27(4):367–8. 10.1016/j.devcel.2013.11.005 24286821

[42] Brideau NJ, Flores HA, Wang J, Maheshwari S, Wang X, Barbash DA. Two Dobzhansky-Muller genes interact to cause hybrid lethality in Drosophila. Science. 2006 11 24;314(5803):1292–5. 17124320 10.1126/science.1133953

[43] Stanke M, Steinkamp R, Waack S, Morgenstern B. AUGUSTUS: a web server for gene finding in eukaryotes. Nucleic Acids Res. 2004 7 1;32(Web Server issue):W309–12. 15215400 10.1093/nar/gkh379 PMC441517

[44] Okonechnikov K, Golosova O, Fursov M; UGENE team. (2012) Unipro UGENE: a unified bioinformatics toolkit. Bioinformatics. 2012 4 15;28(8):1166–7. 10.1093/bioinformatics/bts091 Epub 2012 Feb 24 22368248

[45] Suyama M, Torrents D, Bork P. PAL2NAL: robust conversion of protein sequence alignments into the corresponding codon alignments. Nucleic Acids Res. 34, W609–W612. 16845082 10.1093/nar/gkl315 PMC1538804

[46] Korber B. HIV Signature and Sequence Variation Analysis Computational Analysis of HIV Molecular Sequences, Chapter 4, pages 55–72. Rodrigo Allen G. and Learn Gerald H., eds. Dordrecht, Netherlands: Kluwer Academic Publishers.

[47] Schägger H, von Jagow G. Tricine-sodium dodecyl sulfate-polyacrylamide gel electrophoresis for the separation of proteins in the range from 1 to 100 kDa. Anal Biochem. 1987 11 1;166(2):368–79. 2449095 10.1016/0003-2697(87)90587-2

[48] Sun W, Gao S, Wang L, Chen Y, Wu S, Wang X, et al Microwave-assisted protein preparation and enzymatic digestion in proteomics. Mol Cell Proteomics 2006;5:769–76 16339992 10.1074/mcp.T500022-MCP200

[49] Komonyi O, Pápai G, Enunlu I, Muratoglu S, Pankotai T, Kopitova D, et al DTL, the Drosophila homolog of PIMT/Tgs1 nuclear receptor coactivator-interacting protein/RNA methyltransferase, has an essential role in development. J Biol Chem. 2005 4 1;280(13):12397–404. Epub 2005 Jan 31. 15684427 10.1074/jbc.M409251200

